# In vivo bioluminescence imaging of the intracerebral fibroin-controlled AAV-α-synuclein diffusion for monitoring the central nervous system and peripheral expression

**DOI:** 10.1038/s41598-024-60613-6

**Published:** 2024-04-27

**Authors:** Claire Mazzocco, Coralie Genevois, Qin Li, Evelyne Doudnikoff, Nathalie Dutheil, Thierry Leste-Lasserre, Marie-Laure Arotcarena, Erwan Bezard

**Affiliations:** 1 Institut des Maladies Neurodégénératives, UMR 5293, Univ. de Bordeaux, 33000 Bordeaux, France; 2 Institut des Maladies Neurodégénératives, UMR 5293, CNRS, 33000 Bordeaux, France; 3 VIVOPTIC-TBM-Core Univ Bordeaux, UAR 3427, 33000 Bordeaux, France; 4 Motac Neuroscience, Manchester, M15 6WE UK; 5 INSERM, PUMA, Neurocentre Magendie, U1215, Univ. Bordeaux, 33000 Bordeaux, France

**Keywords:** Neuroscience, Diseases of the nervous system, Parkinson's disease

## Abstract

Among the several animal models of α-synucleinopathies, the well-known viral vector-mediated delivery of wild-type or mutated (A53T) α-synuclein requires new tools to increase the lesion in mice and follow up in vivo expression. To this end, we developed a bioluminescent expression reporter of the human A53T-α-synuclein gene using the NanoLuc system into an AAV2/9, embedded or not in a fibroin solution to stabilise its expression in space and time. We first verified the expression of the fused protein in vitro on transfected cells by bioluminescence and Western blotting. Next, two groups of C57Bl6Jr mice were unilaterally injected with the AAV-NanoLuc-human-A53T-α-synuclein above the *substantia nigra* combined (or not) with fibroin. We first show that the in vivo cerebral bioluminescence signal was more intense in the presence of fibroin. Using immunohistochemistry, we find that the human-A53T-α-synuclein protein is more restricted to the ipsilateral side with an overall greater magnitude of the lesion when fibroin was added. However, we also detected a bioluminescence signal in peripheral organs in both conditions, confirmed by the presence of viral DNA corresponding to the injected AAV in the liver using qPCR.

## Introduction

The hallmarks of Parkinson’s disease (PD), the 2nd most common progressive neurodegenerative disorder, are the selective loss of dopaminergic neurons in the substantia nigra *pars compacta* (SNpc) with proteinaceous inclusions named Lewy bodies and Lewy neurites in surviving neurons^1^. α-synuclein (α-syn) is a major constituent of Lewy bodies and the first disease-causing protein characterized in both sporadic and familial PD^2^. Several α-syn-based animal models of PD have been developed, notably through viral vector-mediated overexpression of the wild-type (WT) or mutated *SNCA* gene that codes for α-syn. Lentiviral or adeno-associated (AAV) viral vector-mediated overexpression of the *SNCA* gene has been successfully conducted in several species, including mice, rats, and non-human primates^3–8^. The largest extent of the lesion is observed in rats compared to mice and non-human primates, with subsequent overt motor and non-motor symptoms in the AAV-α-syn rat^6,9^ but not in mice or non-human primates^3,10^. Beyond possible, but to be fully investigated, inter-species and intra-species differences^3^, the vector type, the AAV serotype, or the titer are obvious variables^11^. We previously reported that titer-equivalent AAV9-mediated mutated human A53T α-syn overexpression induces nigrostriatal neurodegeneration in mice, rats, and non-human primates^3,12–14^ while displaying considerably lesser efficiency in the mouse than in other species^3^.

Among the several factors at work, a greater diffusion within the extracellular space of the different species^15,16^, notably to the hemisphere contralateral to the injection, could play a role and even lead to leakage to the peripheral organs. We thus hypothesised that restricting vector diffusion after injection could lead to (i) greater, more specific lesions of the nigrostriatal pathway and (ii) decreased leakage towards the periphery. Fibroin, a natural protein from Silk cocoon, is used to achieve such restricted diffusion^17^, mostly in optogenetic approaches^18^. To access the in vivo monitoring of α-syn protein localisation and expression, we developed an in vivo bioluminescent expression reporter using the bioluminescent enzyme NanoLuc, efficient for deep-tissue imaging^19^, with NanoLuc and α-syn genes under the control of the synapsin promoter engineered into an AAV9.

We show that mixing AAV with fibroin enables more precise control of transgene expression in the brain, resulting in greater nigral cell loss, with a limited reduction in leakage to peripheral organs, a parameter largely neglected in viral gene transfer in the central nervous system.

## Results

### Expression of the fused NanoLuc-A53Tα-Syn construct

**Figure 1:**
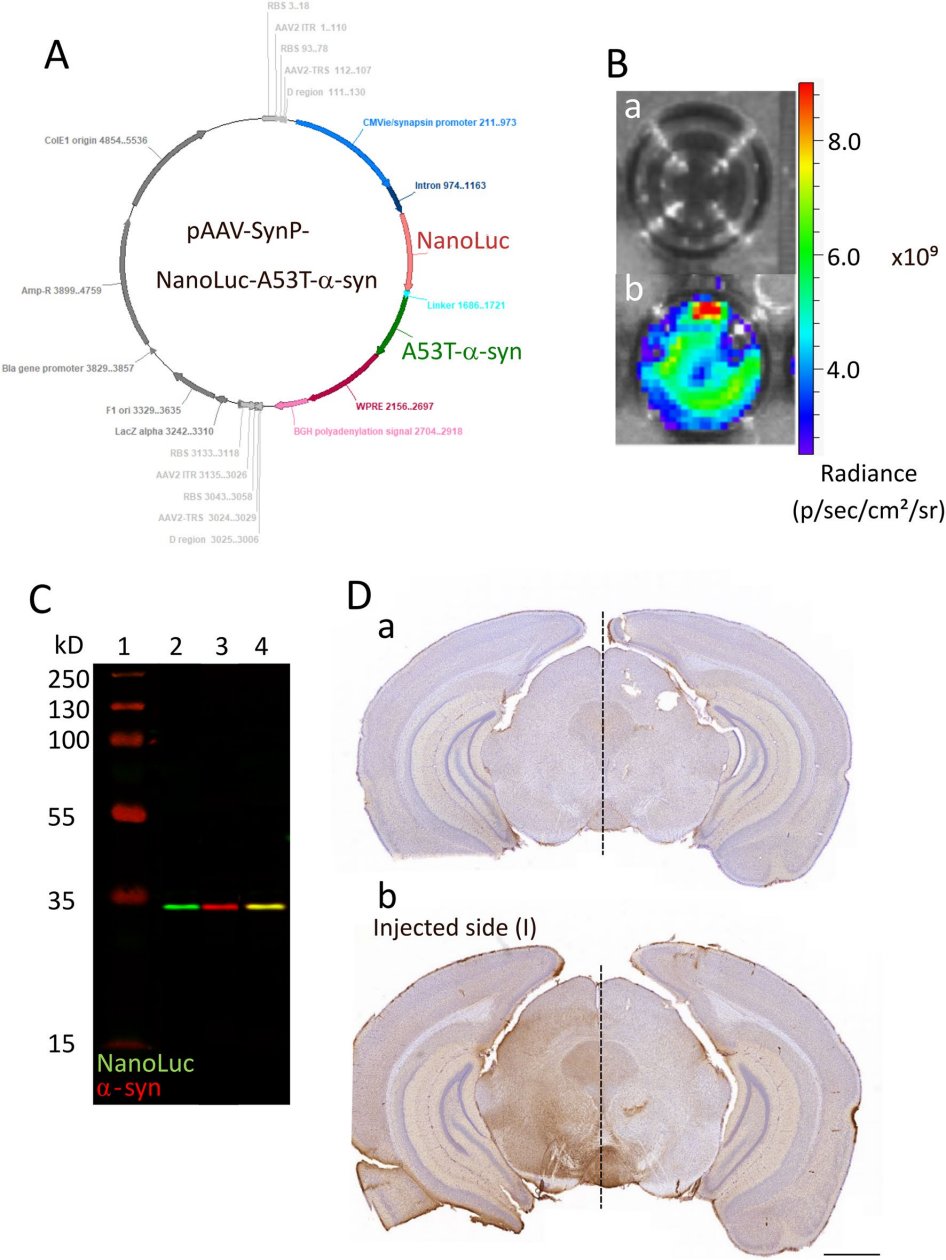
In vitro and in vivo validation of AAV9-SynP-NanoLuc-A53T-a-synuclein expression vector. (**A**) Map of the expression plasmid. (**B**) Bioluminescence imaging of non-transfected (a) or transfected (b) HEK293T cells in p (photon)/sec (second)/cm2/sr (steradian). (**C**) Western blot detection of the fused NanoLuc-A53T-a-synuclein protein. The image shows the acquisition with LiCore's Odyssey fluorescent scanner of the same membrane at wavelengths of 800 nm (anti-Nanoluc) and 700 nm (anti-α-synuclein), and the superposition of the two signals by the scanner. (1) Molecular weight marker (2) anti-NanoLuc detection (3) anti-human-a-synuclein detection (4) Merged signal. (**D**) Representative nigral sections of NanoLuc immunostaining of (a) non-injected and (b) injected mice with the AAV9-SynP-NanoLuc-A53T-a-synuclein (scale bar 1.0 mm).

We first assessed the efficacy of the pAAV-CMVie/SynP-NanoLuc-A53T-α-synuclein-WPRE plasmid (Fig. 1A) to produce NanoLuc-A53Tα-syn protein expression in vitro in HEK293T-transfected cells by bioluminescence imaging and western blotting. Using the Nano-Glo substrate, we were able to detect a bioluminescence signal in transfected cells compared to the non-transfected cells (Fig. 1B), confirming the NanoLuc expression and functionality. Using Western blotting against human α-syn and NanoLuc in the transfected cell extracts, we observed the same protein size band (about 33 kDa) detected at 680 nm (α-syn) and 800 nm (NanoLuc). These data confirm that the NanoLuc bioluminescent detection (Fig. 1B) correlates with the production of the NanoLuc and human α-syn fused protein (Fig. 1C). NanoLuc protein expression was also detected by immunohistochemistry on mesencephalic slices of animals injected with the NanoLuc-α-SynA53T vector compared to the non-injected animals (Fig. 1D). We thus confirmed the expression of the fused protein NanoLuc-A53T-α-synuclein in vitro and in vivo and our ability to follow it using bioluminescence in vitro.

### In vivo NanoLuc-A53T-α-Syn expression

**Figure 2:**
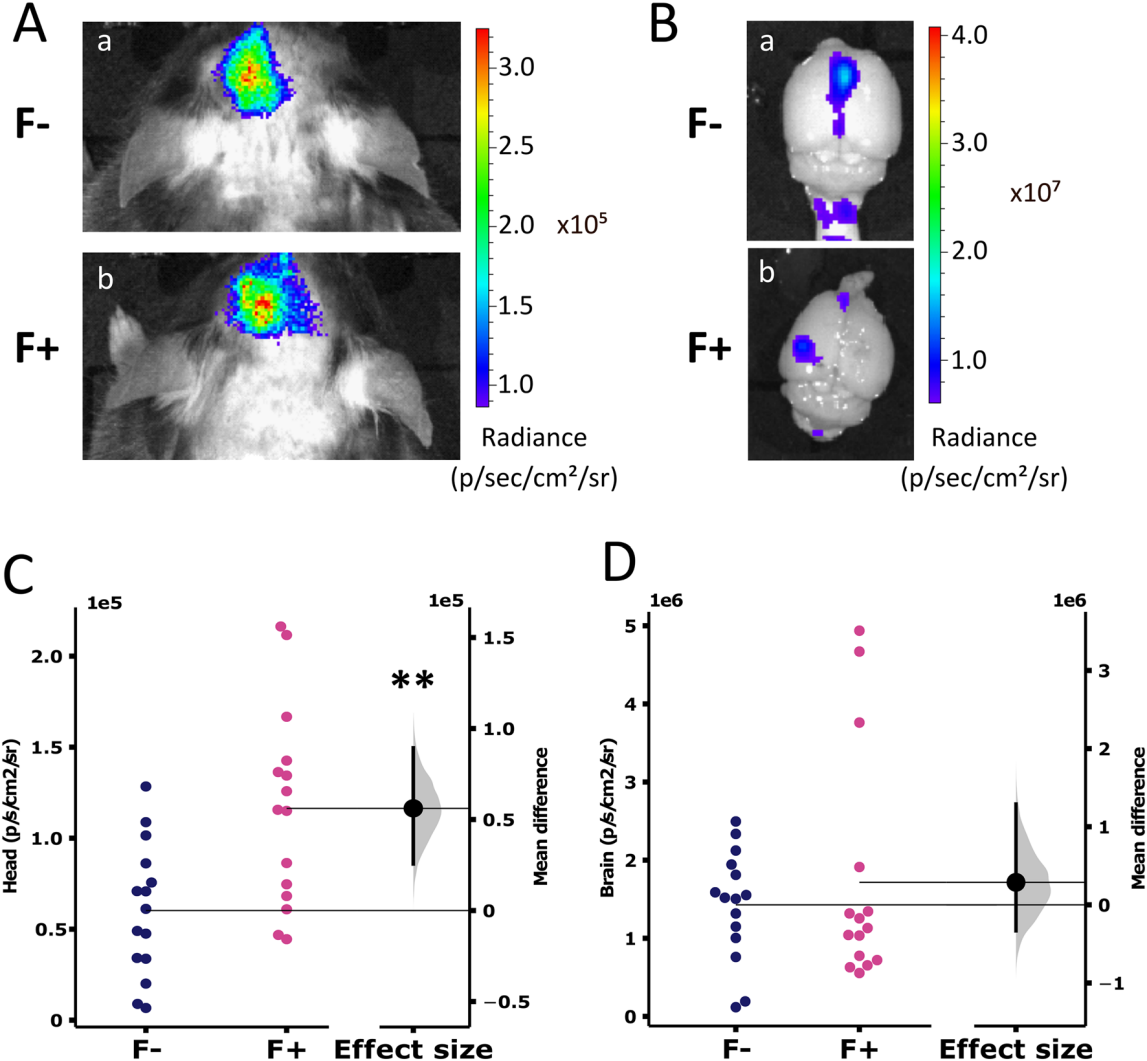
Representative images of in vivo and ex vivo bioluminescence imaging of the head and brain. (**A**) Head and (**B**) brain imaging, of mouse injected with AAV9-SynP-NanoLuc-A53T-a-synuclein without (a) or with (b) fibroin solution. (**C**,**D**) Quantitative analysis of bioluminescence signal detected in the head (**C**) or the brain (**D**) and expressed in p/sec/cm^2^/sr. Horizontal lines represent the mean. Each dot represents one head (**C**) (n = 15, p = 0.0025) or brain (**D**) (n = 15, p = 0.5) of the injected mouse without fibroin (F−, dark blue) and with fibroin (F+, pink). The bootstrapped mean difference with 95% CI (error bar) is shown on the right side of this graph.

In vivo experiments were performed using C57BL/6JRj mice. PD mouse model was induced on gaz anesthetized mouse (2% isoflurane) by the unilateral injection of AAV2/9-CMVie/SynP-NanoLuc-A53Tα-syn-WPRE (2.6 10^13^ GCP/mL) just above the SNpc with or without 4.5% fibroin solution, the silkworm’s cocoon protein. Fused NanoLuc-A53T-α-syn production was then monitored by bioluminescence imaging. A time course pilot study (data not shown) was done to determine the best acquisition timing after substrate injection to the animal and the optimal substrate quantity. Accordingly, 3 min after intraperitoneal injection of 0.088 µmoles of NanoGlo, each animal was anaesthetised and placed into the imager for a 60-s acquisition at 6 min after injection. After a 10-week follow-up, consistent with the installation of the model (at this stage, the detection of the phosphorylated α-synuclein is well established), animals were imaged (Fig. 2A–C) and rapidly euthanised by cervical dislocation for ex vivo imaging of extracted brains (Fig. 2B–D). Bioluminescence signal was detected as early as 2 weeks post-surgery (not shown) and until 10 weeks (Fig. 2). Quantification of radiance (p/s/cm^2^/sr) revealed a brighter bioluminescence signal in the head of animals injected with fibroin (F+), both in vivo (Fig. 2A and C—significant difference) and ex vivo (Fig. 2B and D—trend) than without fibroin (F−). Fibroin would, therefore, allow the maintenance of high expression of the fusion protein.

**Figure 3:**
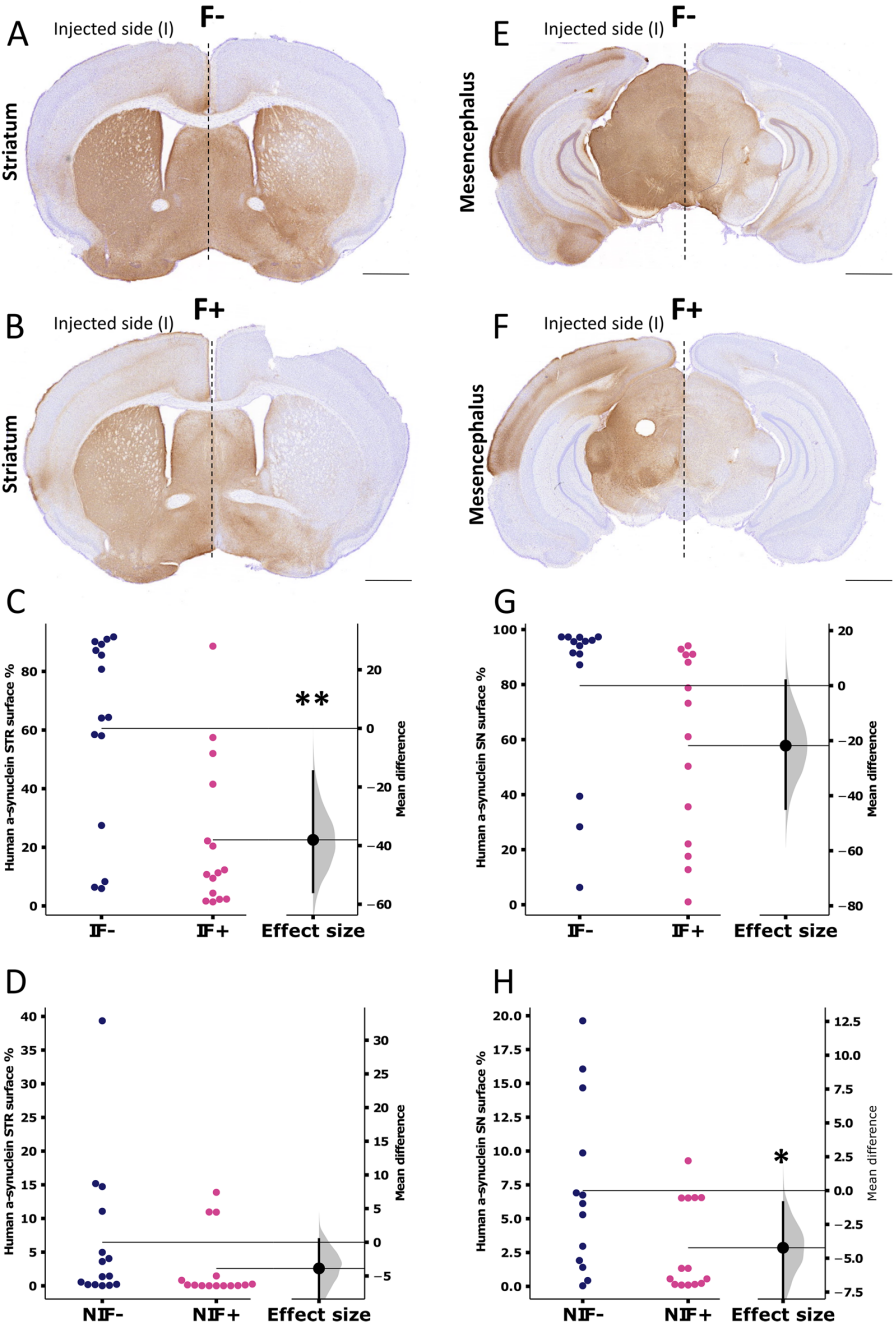
Representative images of α-synuclein immunostaining. Striatal (**A**,**B**) and nigral (**E**,**F**) levels of brain slices 10 weeks after intranigral injection of AAV9-SynP-NanoLuc-A53T-a-synuclein with (F+, **B** and **F**) and without (F−, **A** and **E**) fibroin (scale bar: 2.0 mm). (**C**,**D**,**G**,**H**) Quantitative analysis of α-Synuclein-positive signal expressed in % of surface in the striatum (**C**) (injected side, I, n = 14 p = 0.0015), (**D**) (non-injected side, NI, n = 15 p = 0.209) or nigral (**G**) (injected side I, n = 14 p = 0.0865), (**H**) (non-injected side, NI, n = 13 F− and n = 14 F+ p = 0.0376) slices of the AAV9-SynP-NanoLuc-A53T-a-synuclein mouse without fibroin (F−, dark blue) and with fibroin (F+, pink). Horizontal lines represent the mean. Each dot represents one animal. The bootstrapped mean difference with 95% CI (error bar) is shown on the right side of each graph.

Human A53T-α-syn overexpression was confirmed by immunohistochemistry on the striatal and mesencephalic slices using an anti-human α-syn antibody and quantified as the percentage of the immune-positive stained surface. In both conditions, AAV/NanoLuc-A53T-α-syn, with or without fibroin, resulted in a widespread α-syn expression in the ipsilateral side (injection) and the contralateral side (non-injected) of the striatum and SN (Fig. 3A,B,E,F). The surface of the A53T-α-syn expression was more restricted in the presence of fibroin in the injected side both at the striatal (Fig. 3C) and mesencephalic levels (Fig. 3G), showing a lesser diffusion in the presence of the silk protein (F+). On each side, ispi- or contra-lateral, the surface staining was lower in the presence of fibroin both at the striatal and mesencephalic levels, consistent with a focused expression with limited leakage towards the contralateral side (Fig. 3D–H).

### AAV-NanoLuc-A53T-α-syn injection in the SNpc induces a-Syn phosphorylation

**Figure 4:**
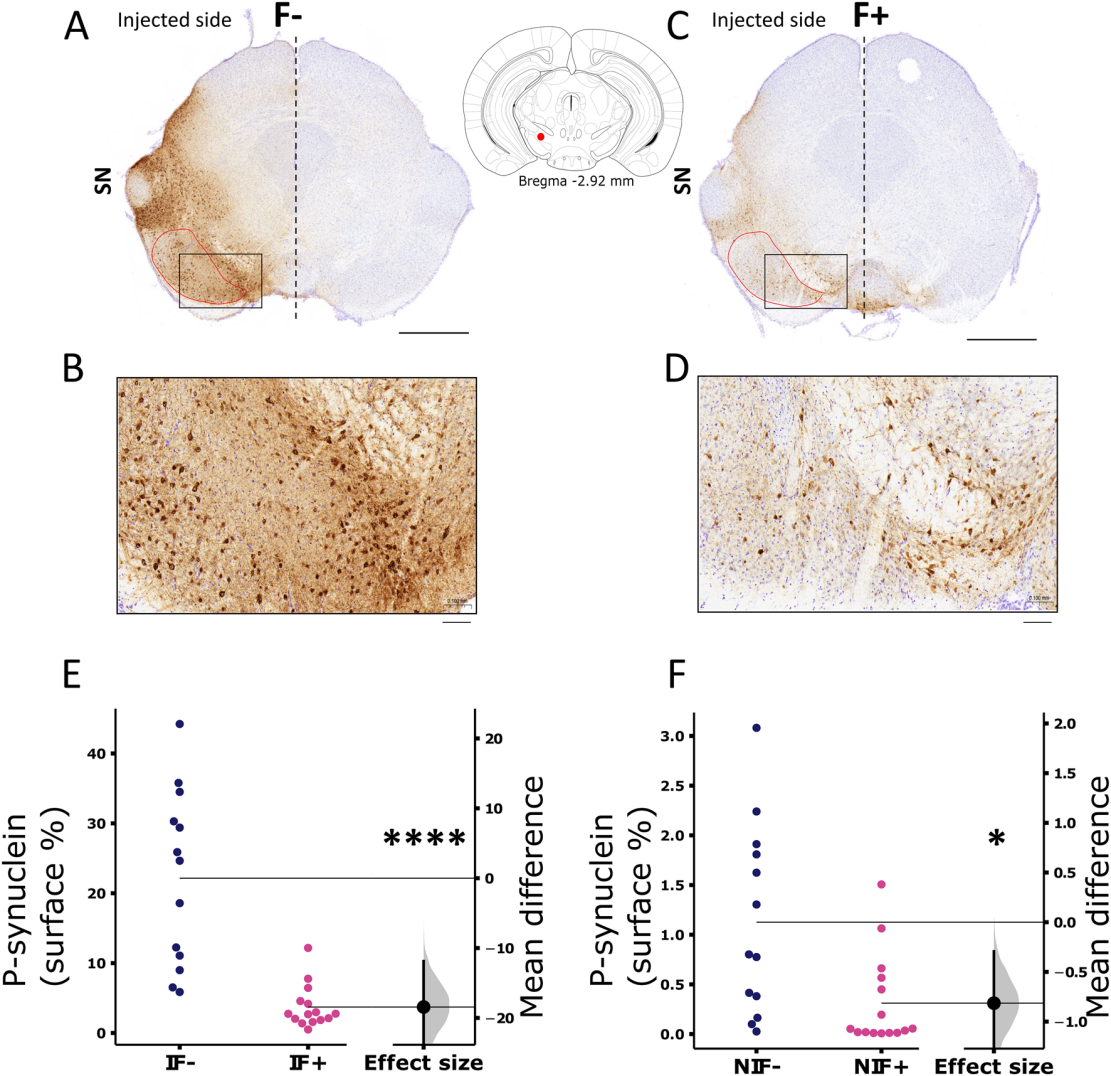
Representative images of S129 phosphorylated α-synuclein immunostaining. Brain mesencephalic slices 10 weeks after intranigral injection of AAV9-SynP-NanoLuc-A53T-a-synuclein without (**A**) or with (**C**) fribroin (scale bars: 1.0 mm), and inset of injection site (red dot) in coronal image on the atlas) and with high magnification (**B** and **D**) (dark inset rectangle fit in (**A**) or (**C**), scale bars: 0.1 mm). (**E**,**F**) Quantitative analysis of S129 phosphorylated α-synuclein-positive signal expressed in % of surface in the SN of injected (I, n = 13 F− and n = 15 F+ p < 0.0001) and non-injected (NI, n = 13 F− and n = 15 F+ p = 0.0495) SN of AAV9-SynP-NanoLuc-A53T-a-synuclein animals without fibroin (F−, dark blue) or with fibroin (F+, pink). Horizontal lines represent the mean. Each dot represents one animal. The bootstrapped mean difference with 95% CI (error bar) is shown for each graph.

We performed an immunostaining against phosphorylated (S129) α-syn (pS129-α-Syn) to investigate the diffusion of the pathology through the SNpc. AAV-NanoLuc-A53T-α-syn vector expression induced strong pS129-α-Syn-positive staining in neurons and neuropils in the injected side compared to the non-injected hemisphere in the mesencephalic slices (Fig. 4A–D). Quantification of the immunostaining surface revealed less extensive staining in the presence of fibroin (F+) in the ipsilateral SNpc (Fig. 4E) and the contralateral side even if the staining was very weak (Fig. 4F), again demonstrating a global focusing effect of the AAV injection with a limited contralateral diffusion in the presence of fibroin.

### AAV-NanoLuc-A53T-α-syn injection in the SNpc leads to dopaminergic neuronal loss

**Figure 5:**
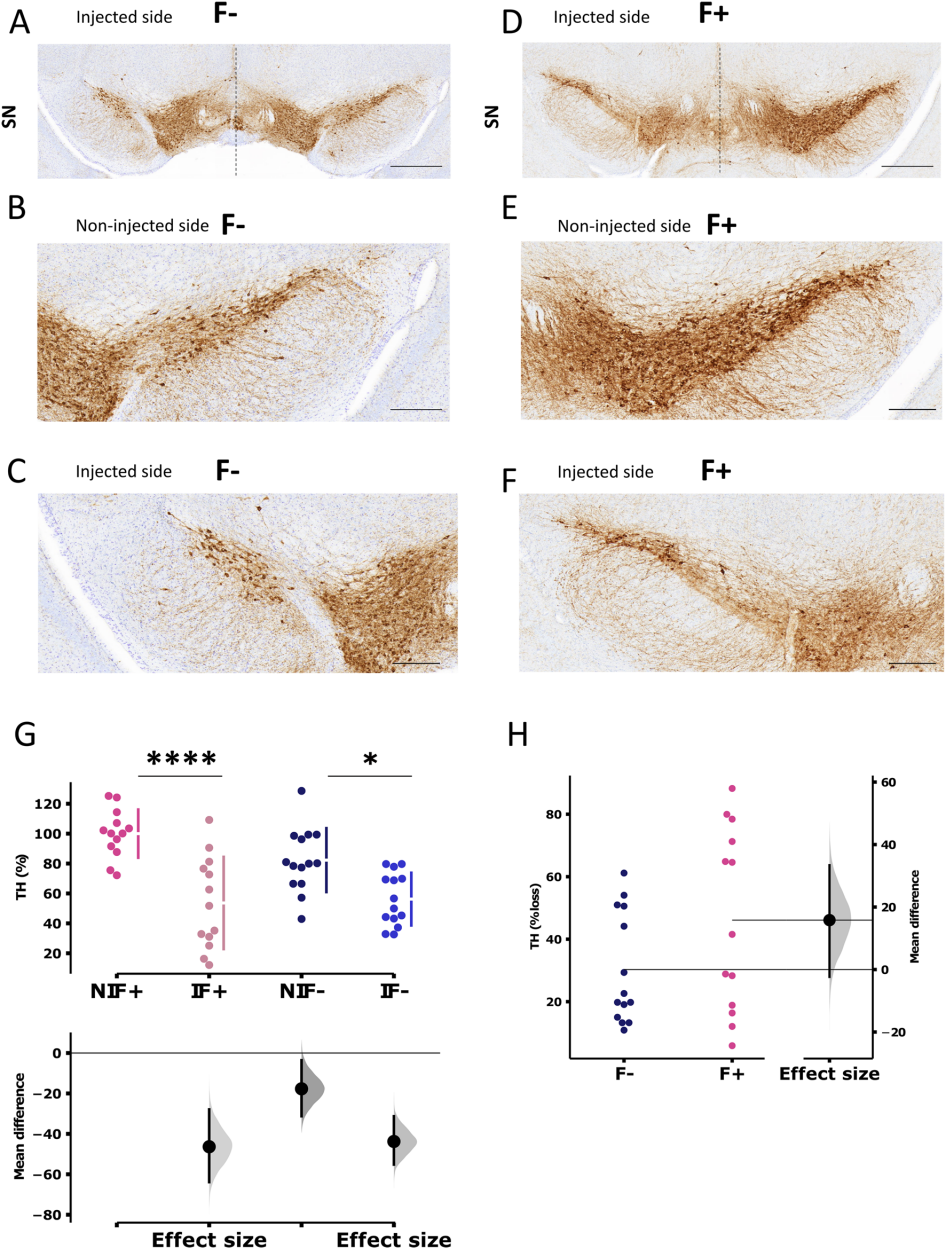
Representative images of Tyrosine Hydroxylase (TH) immunostaining at the mesencephalic level of mice 10 weeks after intranigral injection of AAV9-SynP-NanoLuc-A53T-a-synuclein. Mice were injected with the AAV without fibroin (F−, **A**–**C**) or with (F+, **D**–**F**). (scale bars: 0.5 mm (**A**,**D**) and 0.2 mm (**B**,**C**,**E**,**F**)). (**G**) Total number of TH-positive cells in the non-injected (NI, n = 13 F+ and n = 14 F− p = 0.1736) and injected (I, n = 13 F+ and n = 14 F− p = 0.99) SN of AAV9-SynP-NanoLuc-A53T-a-synuclein animals without fibroin (F−, blue n = 14) or with fibroin (F+, pink n = 13) (p = 0.1, NIF− vs IF− p = 0.0154, NIF+ vs IF+ p < 0.0001). Data were obtained using Visopharm AI deep learning software and expressed as a % of TH cells normalized on NIF. Data are shown as mean ± SD. Each dot represents one animal (**H**) Percentage of TH cell loss in the SN of AAV9-SynP-NanoLuc-A53T-a-synuclein animals with (F+) or without (F−) fibroin. Horizontal lines represent the mean. The bootstrapped mean difference with 95% CI (error bar) is shown for each graph.

Ten weeks post-injection, the extent of nigrostriatal degeneration after AAV/NanoLuc-A53T-α-syn injection was determined by counting the number of tyrosine hydroxylase-immunopositive (TH^+^) neuronal cells within the SNpc using deep learning analysis (Visopharm) in the ipsi- and contralateral sides to the injection, with (F+) or without (F−) fibroin (Fig. 5). The estimated number of TH^+^ dopaminergic neurons in the SNpc was significantly lower on the ipsilateral side with or without fibroin (Fig. 5G) than on the contralateral side, showing that the α-syn-induced lesion was achieved as previously reported^3^. The percentage loss of TH^+^ neurons was not significantly different between the injected sides with (F+) or without (F−) fibroin (Fig. 5H). However, there was a trend towards greater loss in the presence of fibroin (Fig. 5H) (smaller number of TH^+^ neurons counted compared to the control without fibroin). Interestingly, on the contralateral side, the TH^+^ number was lower without fibroin (though not significantly different), showing a few losses of TH^+^ in these conditions (Fig. 5G) and corresponding to a wider spread of the virus injected without fibroin. Such observation raises concerns for studies in which the contralateral side is the reference side to assess the extent of the lesion.

### AAV-NanoLuc-A53T-α-syn leaks to peripheric organs

**Figure 6:**
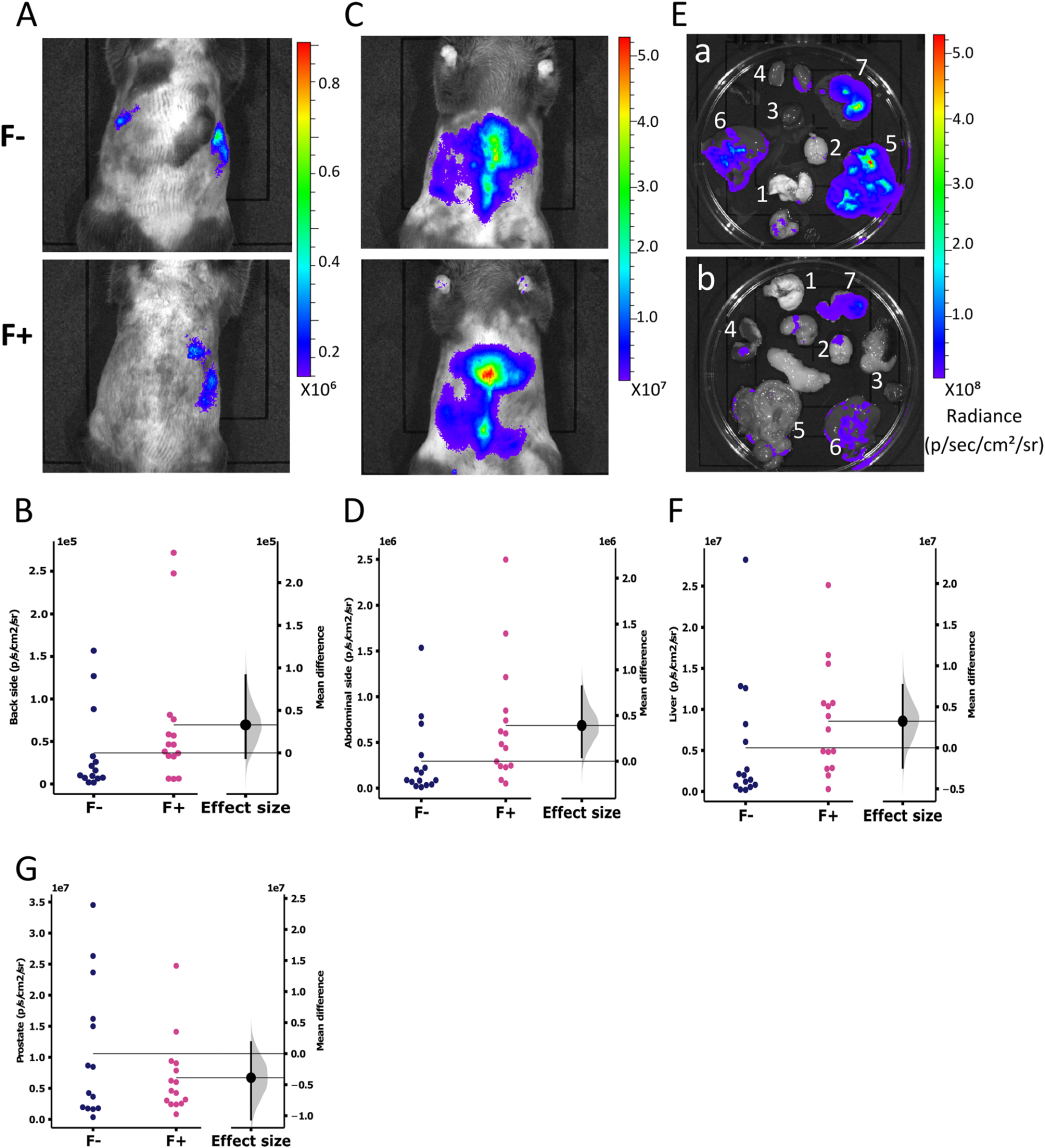
Representative images of in vivo bioluminescence imaging of mice injected with AAV9-SynP-NanoLuc-A53T-a-synuclein without (F−) or with (F+) fibroin. Dorsal (**A**, n = 14 F− and n = 15 F+ p = 0.2) and abdominal (**C**, n = 15 p = 0.065) view of mice. (**E**) Ex vivo bioluminescence imaging of organs from mice injected with AAV9-SynP-NanoLuc-A53T-a-synuclein without (top) or with (bottom) fibroin solution. (1) lung, (2) brain, (3) heart, (4) kidney, (5) intestine, (6) liver, (7) prostate. (**B**,**D**,**F**,**G**) Quantitative analysis of bioluminescence signal detected in body on the back side (**B**) or the abdominal side (D) or in the liver (**F**) (n = 15 p = 0.225) or the prostate (**G**) (n = 14 F− and n = 15 F = p = 0.244) of the AAV9-SynP-NanoLuc-A53T-a-synuclein-injected mouse without fibroin (F−, dark blue) and with fibroin (F+, pink). Data are expressed in p/sec/cm^2^/sr. Horizontal lines represent the mean. Each dot represents an animal. The bootstrapped mean difference with 95% CI (error bar) is shown on the right side of each graph.

NanoLuc-A53T-α-syn production was also functionally monitored by bioluminescence imaging of the whole body. A strong signal was observed on the back and abdominal sides of the animals, with a brighter signal in the presence of fibroin (Fig. 6A–D), suggesting a leakage of the virus expression to the periphery. The regions exhibiting a bioluminescence signal were rapidly dissected, and the organs were imaged 12 to 14 min after the substrate injection (Fig. 6E). The liver and prostate images were analysed (Fig. 6F and G, respectively), showing no significative difference between animals injected with (F+) or without (F−) fibroin. To assess whether the virus disseminated throughout the body and whether the synapsin promoter leaked onto other cells than neurons, we performed qPCR assays to detect the presence of DNA from the injected virus in dissected organs. Five organs were subjected to qPCR. The heart was the negative control for NanoLuc expression, as shown in Fig. 6E. The kidney, intestine, liver, and prostate of 3 animals were analysed because of a significant bioluminescence signal. The AAV DNA was amplified only in the liver with a detection of rate n = 3/3. Virus DNA was not detected under standard qPCR conditions for the other organs, suggesting that leakage was in the blood and not in these organ cells.

## Discussion

The present work demonstrates that mixing AAV with fibroin enables more precise control of transgene expression in the brain, resulting in greater human A53T-α-syn-induced nigral cell loss achieved through preservation of the contralateral mesencephalon, with a marginal reduction in leakage to peripheral organs, a safety translational parameter largely neglected in viral gene transfer in the central nervous system.

The first objective of this work was to ascertain whether the use of fibroin, a protein found in the silkworm cocoon and already used in optogenetic applications to focus the diffusion of reporter probes at the microscope focus point^18^*,* would enhance the A53T-α-syn -induced nigral cell loss. Interestingly, and in complete alignment with its use in optogenetics, fibroin-embedding enabled focus α-syn expression in the ipsilateral side, preventing unwanted contralateral diffusion as shown for both total (Fig. 3) and phosphorylated-syn α-syn (Fig. 4). Such limitation to diffusion resulted in an overall greater lesion of dopamine cells in the SNpc, not through an increased lesioning per se but rather through (i) preserving the contralateral dopamine neurons from the viral vector insult (Fig. 5) and (ii) preventing any unwanted off-target effect of α-syn expression, making the model more efficient. We thus highlighted the relevance of using fibroin to spatially limit a virus expression and achieve the primary goal, which was increasing the lesion window in this mouse model.

The second objective was to monitor human A53T-α-syn expression over time in the brain. We combined the expression of the transgene of interest, A53T-α-syn, to NanoLuc, a luciferase validated for the bioluminescent detection of deep tumours in the brain and disseminated metastases^20^. Although NanoLuc activity generates a blue light (460 nm), it is not optimal for use in deep tissues of living mammals^21^. However, its high brilliance and suitable substrate (NanoGlo) enable imaging of a wide range of mouse organs^20,22^. The in vivo brain acquisition of bioluminescence allows the in vivo selection of animals based on the location and the intensity of bioluminescence, providing a method for decreasing the experimental variability. With n = 15 animals per experimental group, we offered here a relevant cohort to demonstrate the feasibility of such a selection, thanks to the distribution of NanoLuc intensities (Fig. 2A,B).

The third objective was to control for a putative leakage of the AAV to off-target organs. Such leakage occurs at more or less critical levels depending on the AAV serotype and type of promoters. Yet, no systematic methodology other than qPCR after termination exists to answer this question. We used the fused protein of NanoLuc with the gene of interest (regulated by the promoter of choice, here NanoLuc-A53T-α-syn under synapsin control) to monitor the putative leakage to the periphery in living animals. The bioluminescence signal was detected elsewhere than in the head, indicating that NanoLuc was expressed at a distance from the point of intracerebral virus injection. Whole-body bioluminescence imaging (Fig. 6A–D) matched perfectly with ex vivo bioluminescence imaging of the organs (Fig. 6E–G) which corresponds to the qPCR results only on the liver (Fig. 6H). The absence of genomic virus DNA detection in the other, yet bioluminescent, organs might be (i) a consequence of the detection limit or (ii) due to the absence of viral particle but consecutive to accumulation of the leaked fused protein NanoLuc-A53T. AAV9 is generally chosen for intracerebral injection because of its high central nervous system tropism^23^, but it exhibits very good viral transduction in several tissues^24^. This viral capacity of serotype nine and the fact that the synapsin promoter probably has a slight leakage, combined with the sensitive bioluminescence system, revealed a diffusion of the virus and the expression of the fused protein in peripheral organs.

Bioluminescence is a valuable tool for monitoring the biodistribution of gene expression throughout the organism. This noninvasive imaging has become an essential technology for pre-clinical oncology studies to report gene-expressing tumour^25^ cells, providing researchers with a way to track tumour responses to therapies in real-time, longitudinally, in the same animal^26^. This technique was suitable for all research involving gene therapies (e.g. Zika virus detection^27^, vaccination^28^, immune system activation, and macrophage imaging^29^). Also, it represented a rapid and precise tool for screening the biodistribution of new drug candidates. While bioluminescence is an exciting tool for monitoring gene expression, it has limitations, such as the tissue depth (optical imaging is restricted to the distance that photons can travel, i.e. a few millimetres). This shortcoming is improved using a brighter luciferase, NanoLuc, which allows deeper tissues to be imaged. Brighter substrates are under development, which will extend the technology applications.

Imaging protein aggregates that are the pathological landmarks of neurodegenerative diseases is a possible development that would require further studies. The AAV-A53T-α-syn model does not produce Lewy body-like intracytoplasmic aggregates but, at best, protein kinase-resistant puncta of phospho-α-syn, i.e. small structures essentially composed of α-syn. In contrast, Lewy body-like inclusions would contain hundreds of proteins and organelles^30–32^. Besides the inadequacy of the present model (that models the overexpression of monomeric synuclein seen in familial PD with duplication, triplication or quadruplication of the SNCA gene^33^), the question remains. Mixing α-syn preformed fibrils^34,35^ or PD patients’ brain extracts^36–38^ with an AAV-nanoluc (or directly with small amounts of nanoluc itself) should be tested to hopefully indirectly image aggregates in the living animal brain.

## Conclusions

Our data have several consequences: (i) Mixing AAV with fibroin ensures accurate control of transgene expression. (ii) In vivo NanoLuc-human-A53T-α-syn protein expression tracking by bioluminescence imaging would enable proper animal selection in preclinical testing of therapeutic strategies, (iii) first by allowing post-surgery animal selection and (iv) by possibly providing an in vivo monitored end-point of success (or failure) of the tested therapy. These four results make the use of.

## Methods

### Plasmid constructs and AAV vector production

The pAAV-CMVie/SynP-NanoLuc-A53T-α-synuclein-WPRE plasmid was made by replacing the α-synuclein coding region of pAAV-CMVie/Syn-A53T-α-synuclein-WPRE with a nano luciferase (NanoLuc)-A53T-α-synuclein fragment (gene strand DNA fragment synthesised by Eurofins Genomics, Ebersberg, Germany). Recombinant AAV9 vectors were produced by polyethyleneimine (PEI) mediated triple transfection of low passage HEK-293T /17 cells (ATCC; cat number CRL-11268). The AAV expression plasmid pAAV2-CMVie/hSyn-NanoLuc-synA53T-WPRE was co-transfected with the adeno helper pAd Delta F6 plasmid (Penn Vector Core, cat # PL-F-PVADF6) and AAV Rep Cap pAAV2/9 plasmid (Penn Vector Core, cat # PLT-PV008). AAV vectors were purified as previously described^3,12,39,40^.

### Quantitative real-time PCR (qPCR)

Genomic DNA (gDNA) was extracted from tissues using a QIAamp DNA mini kit (Qiagen). QPCR was performed using a LightCycler^®^ 480 Real-Time PCR System (Roche, Meylan, France). QPCR reactions were done in duplicate for each sample, using transcript-specific primers, gDNA (1ng), and LightCycler 480 SYBR Green I Master (Roche) in a final volume of 10 μl. Actin beta (Actb) was used as a control gene for gDNA quantification. Primer sequences are reported in Table 1.

**Table 1:** qPCR primer sequences.

Gene	GenBank ID	Forward sequence (5ʹ–3ʹ)	Reverse sequence (5ʹ–3ʹ)
AAV	AH002306	GGAACCCCTAGTGATGGAGTT	CGGCCTCAGTGAGCGA
Actb (gDNA)	AC144818	GCCCTAGGCACCAGGTAAGTG	GCATCGATCCCCAAGAAAAC

### Cell culture and transfection

HEK293T cells were cultured into 6- or 48-well plates for western blotting or imaging in complete DMEM (MERCK Sigm-Aldrich). Transfection was done the day after the seeding using a standard commercial protocol for PEI (MERCK Sigma-Aldrich), using 4 µg per well (6-well plate) or 0.5 µg of DNA (48-well plate). The protein expression was assessed 48 h after the transfection.

### Western blotting

Cells were harvested by trypsinization for 2 min at 37 °C, rinsed with PBS, and centrifugated. The pellet was resuspended in SDS extraction buffer (50 mM Tris pH 7.6, 150 mM NaCl, 1% Triton-X-100, 0.5% Na-deoxycholate, 1% SDS) and then stored at − 20 °C until use. After BCA (Pierce) quantification, 20 µg of protein were loaded onto 12% SDS–polyacrylamide gel, separated by electrophoresis, and transferred to nitrocellulose membrane as described in^12^. Incubation of the primary antibodies was performed overnight at 4 °C with rabbit anti-human α-synuclein (Abcam MJFR1 ab138501, RRID: AB_2537217, 1/5000) and mouse anti-NanoLuc (Promega, N7000, RRID: AB_3095534, 1/1000) antibodies on the same membrane. Appropriate secondary antibodies coupled to IRDye 680 (Li-Core 922-32210, RRID: AB_10956166, 1/5000) or 800 nm (Li-Core 926-68071, RRID: AB_2687825, 1/5000) were revealed also on the same membrane using an Odyssey Li-Core scanner as previously described^41^.

### Fibroin preparation

Briefly, 5 g of silkworm cocoons were cut into small pieces and boiled in 2 L of Na_2_Co_3_ (0.02 M), washed 3 times in ultrapure water, squeezed, and dried overnight^17^. On day 2, the silk fibers were dissolved in LiBr solution (9.3 M, 0.25 g/mL (w/v)) for 4 h at 60 °C. Then, the solution was dialyzed against ultra-pure water 6-time changes for 48 h. Finally, the solution was centrifuged twice (20 min, 12,700 *g* at 4 °C) and used directly for stereotactic injection or lyophilized for further storage at room temperature.

### Animals and surgical procedures

Six-week-old wild-type C57BL/6Jr mice were purchased from Janvier laboratories and reared at the University of Bordeaux animal facilities. Animals were maintained in standard conditions under a 12-h light/dark cycle with water and food provided ad libitum and acclimatised for 2 weeks before starting the experiments. For surgery, anaesthetised (isoflurane 2%) mice received 2 µL of AAV9-CMVie/SynP-NanoLuc-A53T-α-synuclein-WPRE (concentration: 2.6 10^13^ GCP/ml) in sterile PBS or 4.5% of fibroin solution, by stereotactic delivery (400 nL/min rate) to the region immediately above the left substantia nigra AP-2.9/ML − 1.3/DV − 4.5) as previously described^3^.

### Bioluminescence imaging (BLI)

Imaging was performed at the Vivoptic platform (University of Bordeaux, Bordeaux) using a Lumina III imaging system (Perkin Elmer Inc., Boston, MA, USA). HEK293T transfected cells were imaged (0.5 s) by bioluminescence 6 min after adding NanoGlo substrate (25 µM in 100 μL PBS; Promega).

For in vivo experiments and to determine the optimal experimental conditions, various NanoGlo substrate FFZ (Promega, Madison, WI, USA) quantities were injected intraperitoneally into the mice (0.35, 0.175, 0.088, and 0.044 µmoles). Three minutes after the substrate injection, mice were sedated, and bioluminescence acquisitions were performed (1 min, 4 × 4 binning) at 4, 6, 8, 10, and 12 min post-injection. Finally, mice received an intraperitoneal injection of 0.088 µmoles of NanoGlo substrate FFZ (Promega, Nano-Glo^®^ Fluorofurimazine FFz) for all the bioluminescence acquisitions and were sedated 3 min later. Bioluminescence images and photographs (100 ms) were taken 6 min after the substrate injection. Image quantification was done using Living Image software (Perkin Elmer). At 10 weeks post-surgery, mice were euthanized by cervical dislocation, and organs were then rapidly retrieved for BLI.

### Histopathological analysis

Brain immunostaining anti-NanoLuc (1:1000 N7000 Promega), TH (1:5000 clone EP1532Y, ab137869 Abcam, RRID: AB_2801410), human α-synuclein (1:1000 clone MJFR1, ab138501 Abcam) or human phosphorylated Ser129 α-synuclein (1:5000, EP1536Y, ab51253, Abcam, RRID:AB_869973) were performed as previously described^42^ for each animal, on striatal sections and every 5 midbrain sections spanning the entire rostro caudal SNpc. Immunoreactions were revealed by an anti–species dependent peroxidase EnVisionTM system (DAKO, mouse K4001 and rabbit K4003) followed by DAB visualization.

Human α-synuclein-positive immunostaining was quantified with a Leica DM-6000B microscope and the Mercator Pro software (Explora Nova) as previously described^3^. Briefly, regions of interest (ROIs) were drawn as a surface mask on the hemi striatal and mesencephalic sections. Six brain sections were analyzed to obtain an average representation of the rostrocaudal viral diffusion (for the striatum and substantia nigra).

Nigral TH-positive cells were counted on 6 sections using the Visiopharm software. Briefly, the SN area was delineated for each hemisphere (left and right). The software was previously trained using a deep learning network to detect TH-positive cells manually drawn in the tissue. Once validated on a training batch, the deep learning analysis was launched on the SN to obtain the total number of TH-positive cells detected in the SN. The S129-phospho-α-synuclein positive-immunostaining was quantified using a threshold detection macro on Visiopharm applied in a surface mask of 6 delineated hemi-mesencephalic slices of the injected (I) or non-injected (NI) side without (F−) or with (F+) fibroin.

### Statistical analysis

For the experiments, comparisons between means were carried out by using the Student T test or two-way ANOVA and followed, when appropriate, by pairwise comparison between means by Tukey post hoc analysis. Statistical analyses were performed with GraphPad Prism 9.2.0 (GraphPad Software, Inc., San Diego, CA). Statistical significance was set at a p-value p < 0.05.

The debate about the need to move beyond p-value is raging and data must be analyzed further with estimation graphics emphasizing the effect size^43^. Therefore, we used an estimation graphic called ‘Gardner–Altman plot’ to present the data as previously shown^44^. This plot uses two graphs. The left graph presents data from different groups as scatter plots showing the observed values along with the above-defined descriptive statistics (mean ± SEM). The right or lower graph displays the effect size by presenting the distribution of the difference between the groups using resampled distributions of observed data. Horizontally aligned with the mean of the test group, the mean difference is indicated by the black circle. The black vertical line illustrates the 95% CI of the mean difference.

### Ethics approval

Experiments were performed following the European Union directive (2010/63/EU) on protecting animals used for scientific purposes. They were approved by the Ethical Committee of Bordeaux University (CEEA 50, France) and the Ministry of Education and Research under license number #34302-2021120616221554-v11. The study is reported in accordance with ARRIVE guidelines (https://arriveguidelines.org).

### Supplementary Information

Supplementary Information 1.

Supplementary Information 2.

Supplementary Information 3.

## Data Availability

The data and materials presented in this study are available on request from the corresponding author and the raw data are available as supplementary file.
