# Supplementary figures and images for: In vivo bioluminescence imaging of the intracerebral fibroin-controlled AAV-α-synuclein diffusion for monitoring the central nervous system and peripheral expression
Source: Sci Rep. 2024 Apr 27;14:9710. doi: 10.1038/s41598-024-60613-6 (PMC11055870; doi:10.1038/s41598-024-60613-6)

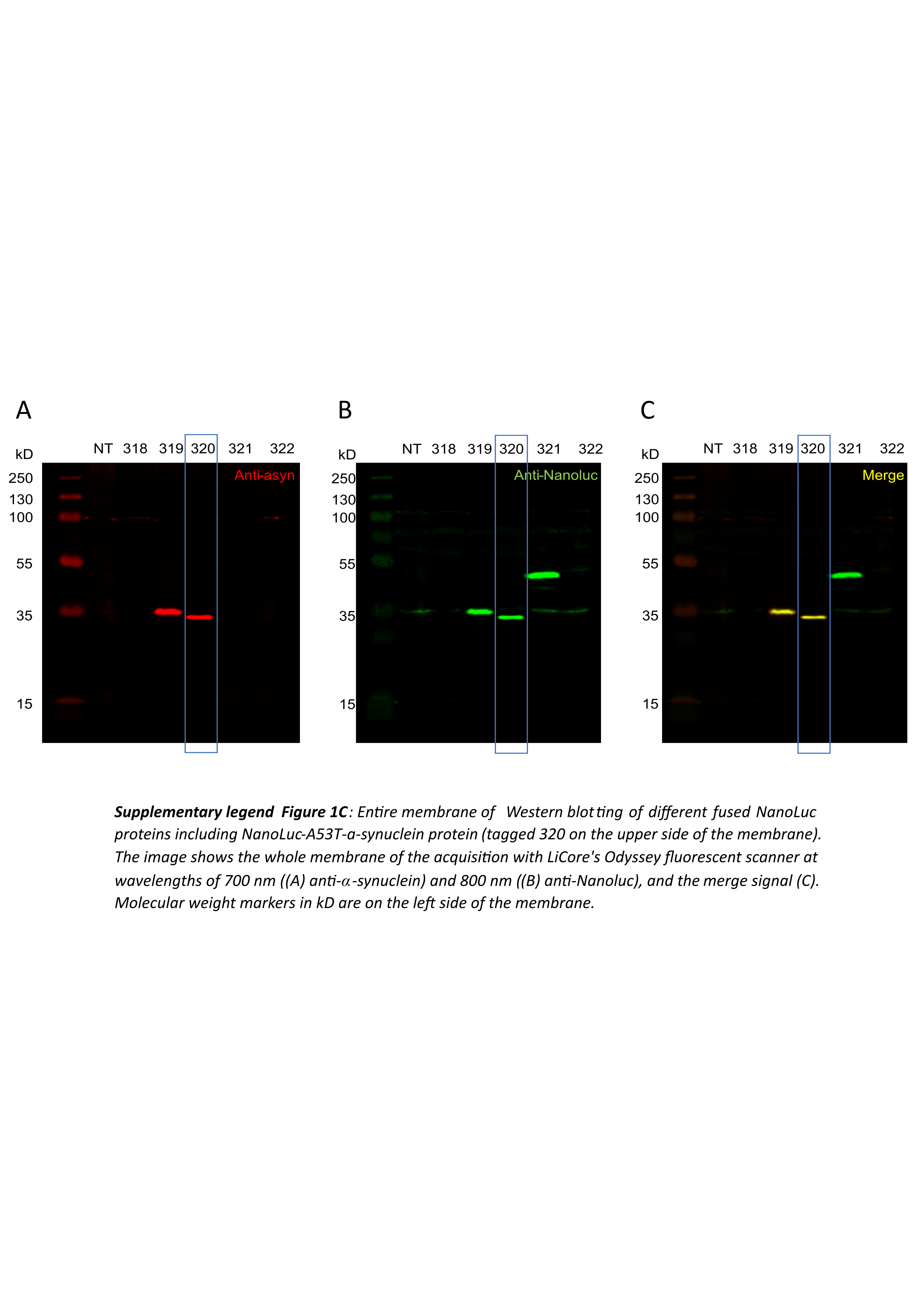

Supplement: Supplementary file 2 — Supplementary Information 2. [file 41598_2024_60613_MOESM2_ESM.tiff]
