# Supplementary material for: In vivo bioluminescence imaging of the intracerebral fibroin-controlled AAV-α-synuclein diffusion for monitoring the central nervous system and peripheral expression
Source: Sci Rep. 2024 Apr 27;14:9710. doi: 10.1038/s41598-024-60613-6 (PMC11055870; doi:10.1038/s41598-024-60613-6)
Supplement: Supplementary file 3 — Supplementary Information 3. [file 41598_2024_60613_MOESM3_ESM.pdf]

Tue Mar 12, 2024 13:40 +0100
pAAV-CMVie_SynP-NanoLuc-A53T-synuclein-WPRE.str
Text Map

```
             10        20        30        40        50        60        70        80        90       100
         *    *    *    *    *    *    *    *    *    *    *    *    *    *    *    *    *    *    *    *
   1 ctgcgcgctcgctcgctcactgaggccgcccgggcaaagcccgggcgtcgggcgacctttggtcgcccggcctcagtgagcgagcGAGCGCGCAGAGAGG 100
     >>>>>>>>>>>>>>>>>>>>>>>>>>>>>>>>>>>>>>>>>>>>>>>>>>>>>>>>>>>>>>>>>>>>>>>>>>>>>>>>>>>>>>>>>>>>>>>>>>>
     ITR

            110       120       130       140       150       160       170       180       190       200
         *    *    *    *    *    *    *    *    *    *    *    *    *    *    *    *    *    *    *    *
 101 GAGTGGCCAACTCCATCACTAGGGGTTCCTTGTAGTTAATGATTAACCCGCCATGCTACTTATCTACGTAGCCATGCTCTAGGAAGATCGGAATTCGCCC 200
     >>>>>>>>>>
     ITR

            210       220       230       240       250       260       270       280       290       300
         *    *    *    *    *    *    *    *    *    *    *    *    *    *    *    *    *    *    *    *
 201 TTAAGCTAGATAGTTATTAATAGTAATCAATTACGGGGTCATTAGTTCATAGCCCATATATGGAGTTCCGCGTTACATAACTTACGGTAAATGGCCCGCC 300
               >>>>>>>>>>>>>>>>>>>>>>>>>>>>>>>>>>>>>>>>>>>>>>>>>>>>>>>>>>>>>>>>>>>>>>>>>>>>>>>>>>>>>>
               CMV enhancer/Synapsin promoter

            310       320       330       340       350       360       370       380       390       400
         *    *    *    *    *    *    *    *    *    *    *    *    *    *    *    *    *    *    *    *
 301 TGGCTGACCGCCCAACGACCCCCGCCCATTGACGTCAATAATGACGTATGTTCCCATAGTAACGCCAATAGGGACTTTCCATTGACGTCAATGGGTGGAG 400
     >>>>>>>>>>>>>>>>>>>>>>>>>>>>>>>>>>>>>>>>>>>>>>>>>>>>>>>>>>>>>>>>>>>>>>>>>>>>>>>>>>>>>>>>>>>>>>>>>>>
     CMV enhancer/Synapsin promoter

            410       420       430       440       450       460       470       480       490       500
         *    *    *    *    *    *    *    *    *    *    *    *    *    *    *    *    *    *    *    *
 401 TATTTACGGTAAACTGCCCACTTGGCAGTACATCAAGTGTATCATATGCCAAGTACGCCCCCTATTGACGTCAATGACGGTAAATGGCCCGCCTGGCATT 500
     >>>>>>>>>>>>>>>>>>>>>>>>>>>>>>>>>>>>>>>>>>>>>>>>>>>>>>>>>>>>>>>>>>>>>>>>>>>>>>>>>>>>>>>>>>>>>>>>>>>
     CMV enhancer/Synapsin promoter

            510       520       530       540       550       560       570       580       590       600
         *    *    *    *    *    *    *    *    *    *    *    *    *    *    *    *    *    *    *    *
 501 ATGCCCAGTACATGACCTTATGGGACTTTCCTACTTGGCAGTACATCTACGTATTAGTCATCGCTATTACCATGGCACCTGCAGAGGGCCCTGCGTATGA 600
     >>>>>>>>>>>>>>>>>>>>>>>>>>>>>>>>>>>>>>>>>>>>>>>>>>>>>>>>>>>>>>>>>>>>>>>>>>>>>>>>>>>>>>>>>>>>>>>>>>>
     CMV enhancer/Synapsin promoter

            610       620       630       640       650       660       670       680       690       700
         *    *    *    *    *    *    *    *    *    *    *    *    *    *    *    *    *    *    *    *
 601 GTGCAAGTGGGTTTTAGGACCAGGATGAGGCGGGGTGGGGGTGCCTACCTGACGACCGACCCCGACCCACTGGACAAGCACCCAACCCCCATTCCCCAAA 700
     >>>>>>>>>>>>>>>>>>>>>>>>>>>>>>>>>>>>>>>>>>>>>>>>>>>>>>>>>>>>>>>>>>>>>>>>>>>>>>>>>>>>>>>>>>>>>>>>>>>
     CMV enhancer/Synapsin promoter

            710       720       730       740       750       760       770       780       790       800
         *    *    *    *    *    *    *    *    *    *    *    *    *    *    *    *    *    *    *    *
 701 TTGCGCATCCCCTATCAGAGAGGGGGAGGGGAAACAGGATGCGGCGAGGCGCGTGCGCACTGCCAGCTTCAGCACCGCGGACAGTGCCTTCGCCCCCGCC 800
     >>>>>>>>>>>>>>>>>>>>>>>>>>>>>>>>>>>>>>>>>>>>>>>>>>>>>>>>>>>>>>>>>>>>>>>>>>>>>>>>>>>>>>>>>>>>>>>>>>>
     CMV enhancer/Synapsin promoter

            810       820       830       840       850       860       870       880       890       900
         *    *    *    *    *    *    *    *    *    *    *    *    *    *    *    *    *    *    *    *
 801 TGGCGGCGCGCGCCACCGCCGCCTCAGCACTGAAGGCGCGCTGACGTCACTCGCCGGTCCCCCGCAAACTCCCCTTCCCGGCCACCTTGGTCGCGTCCGC 900
     >>>>>>>>>>>>>>>>>>>>>>>>>>>>>>>>>>>>>>>>>>>>>>>>>>>>>>>>>>>>>>>>>>>>>>>>>>>>>>>>>>>>>>>>>>>>>>>>>>>
     CMV enhancer/Synapsin promoter

            910       920       930       940       950       960       970       980       990      1000
         *    *    *    *    *    *    *    *    *    *    *    *    *    *    *    *    *    *    *    *
 901 GCCGCCGCCGGCCCAGCCGGACCGCACCACGCGAGGCGCGAGATAGGGGGGCACGGGCGCGACCATCTGCGCTGCGGCCGCACTAGTTGGTCGTGAGGCA 1000
     >>>>>>>>>>>>>>>>>>>>>>>>>>>>>>>>>>>>>>>>>>>>>>>>>>>>>>>>>>>>>>>>>>>>>>>>>>>>>>>>>>>>>>>>>>>>>>>>>>>
     CMV enhancer/Synapsin promoter

           1010      1020      1030      1040      1050      1060      1070      1080      1090      1100
         *    *    *    *    *    *    *    *    *    *    *    *    *    *    *    *    *    *    *    *
1001 CTGGGCAGGTAAGTATCAAGGTTACAAGACAGGTTTAAGGAGACCAATAGAAACTGGGCTTGTCGAGACAGAGAAGACTCTTGCGTTTCTGATAGGCACC 1100
     >>>>>>>>>>>>>>>>>>>>>>>>>>>>>>>>>>>>>>>>>>>>>>>>>>>>>>>>>>>>>>>>>>>>>>>>>>>>>>>>>>>>>>>>>>>>>>>>>>>
     CMV enhancer/Synapsin promoter

           1110      1120      1130      1140      1150      1160      1170      1180      1190      1200
         *    *    *    *    *    *    *    *    *    *    *    *    *    *    *    *    *    *    *    *
1101 TATTGGTCTTACTGACATCCACTTTGCCTTTCTCTCCACAGGTGTCCAGGCGGCCGCTCTAGAGGATCTACCATGGTCTTCACACTCGAAGATTTCGTTG 1200
```

```
                                                                             M  V  F  T  L  E  D  F  V  G
                                                                             1
                                                                             >>>>>>>>>>>>>>>>>>>>>>>>>>>>
                                                                             Nanoluc
     >>>>>>>>>>>>>>>>>>>>>>>>>>>>>>>>>>>>>>>>>>>>>>>>>>>>>>>>>>>>>>>
     CMV enhancer/Synapsin promoter

            1210      1220      1230      1240      1250      1260      1270      1280      1290      1300
         *    *    *    *    *    *    *    *    *    *    *    *    *    *    *    *    *    *    *    *
1201 GGGACTGGCGACAGACAGCCGGCTACAACCTGGACCAAGTCCTTGAACAGGGAGGTGTGTCCAGTTTGTTTCAGAATCTCGGGGTGTCCGTAACTCCGAT 1300
       D  W  R  Q  T  A  G  Y  N  L  D  Q  V  L  E  Q  G  G  V  S  S  L  F  Q  N  L  G  V  S  V  T  P  I
     11
     >>>>>>>>>>>>>>>>>>>>>>>>>>>>>>>>>>>>>>>>>>>>>>>>>>>>>>>>>>>>>>>>>>>>>>>>>>>>>>>>>>>>>>>>>>>>>>>>>>>>
     Nanoluc

            1310      1320      1330      1340      1350      1360      1370      1380      1390      1400
         *    *    *    *    *    *    *    *    *    *    *    *    *    *    *    *    *    *    *    *
1301 CCAAAGGATTGTCCTGAGCGGTGAAAATGGGCTGAAGATCGACATCCATGTCATCATCCCGTATGAAGGTCTGAGCGGCGACCAAATGGGCCAGATCGAA 1400
      Q  R  I  V  L  S  G  E  N  G  L  K  I  D  I  H  V  I  I  P  Y  E  G  L  S  G  D  Q  M  G  Q  I  E
     44
     >>>>>>>>>>>>>>>>>>>>>>>>>>>>>>>>>>>>>>>>>>>>>>>>>>>>>>>>>>>>>>>>>>>>>>>>>>>>>>>>>>>>>>>>>>>>>>>>>>>>
     Nanoluc

            1410      1420      1430      1440      1450      1460      1470      1480      1490      1500
         *    *    *    *    *    *    *    *    *    *    *    *    *    *    *    *    *    *    *    *
1401 AAAATTTTTAAGGTGGTGTACCCTGTGGATGATCATCACTTTAAGGTGATCCTGCACTATGGCACACTGGTAATCGACGGGGTTACGCCGAACATGATCG 1500
     K  I  F  K  V  V  Y  P  V  D  D  H  H  F  K  V  I  L  H  Y  G  T  L  V  I  D  G  V  T  P  N  M  I  D
     77
     >>>>>>>>>>>>>>>>>>>>>>>>>>>>>>>>>>>>>>>>>>>>>>>>>>>>>>>>>>>>>>>>>>>>>>>>>>>>>>>>>>>>>>>>>>>>>>>>>>>>
     Nanoluc

            1510      1520      1530      1540      1550      1560      1570      1580      1590      1600
         *    *    *    *    *    *    *    *    *    *    *    *    *    *    *    *    *    *    *    *
1501 ACTATTTCGGACGGCCGTATGAAGGCATCGCCGTGTTCGACGGCAAAAAGATCACTGTAACAGGGACCCTGTGGAACGGCAACAAAATTATCGACGAGCG 1600
       Y  F  G  R  P  Y  E  G  I  A  V  F  D  G  K  K  I  T  V  T  G  T  L  W  N  G  N  K  I  I  D  E  R
     111
     >>>>>>>>>>>>>>>>>>>>>>>>>>>>>>>>>>>>>>>>>>>>>>>>>>>>>>>>>>>>>>>>>>>>>>>>>>>>>>>>>>>>>>>>>>>>>>>>>>>>
     Nanoluc

            1610      1620      1630      1640      1650      1660      1670      1680      1690      1700
         *    *    *    *    *    *    *    *    *    *    *    *    *    *    *    *    *    *    *    *
1601 CCTGATCAACCCCGACGGCTCCCTGCTGTTCCGAGTAACCATCAACGGAGTGACCGGCTGGCGGCTGTGCGAACGCATTCTGGCGGGATCCGCTGGCTCC 1700
      L  I  N  P  D  G  S  L  L  F  R  V  T  I  N  G  V  T  G  W  R  L  C  E  R  I  L  A
     144
     >>>>>>>>>>>>>>>>>>>>>>>>>>>>>>>>>>>>>>>>>>>>>>>>>>>>>>>>>>>>>>>>>>>>>>>>>>>>>
     Nanoluc
                                                                                          G  S  A  G  S
                                                                                          1
                                                                                          >>>>>>>>>>>>>>>
                                                                                          linker

            1710      1720      1730      1740      1750      1760      1770      1780      1790      1800
         *    *    *    *    *    *    *    *    *    *    *    *    *    *    *    *    *    *    *    *
1701 GCTGCTGGTTCTGGCGAATTCATGGATGTATTCATGAAAGGACTTTCAAAGGCCAAGGAGGGAGTTGTGGCTGCTGCTGAGAAAACCAAACAGGGTGTGG 1800
                          M  D  V  F  M  K  G  L  S  K  A  K  E  G  V  V  A  A  A  E  K  T  K  Q  G  V  A
                          1
                          >>>>>>>>>>>>>>>>>>>>>>>>>>>>>>>>>>>>>>>>>>>>>>>>>>>>>>>>>>>>>>>>>>>>>>>>>>>>>>>
                          A53T-?-syn
     A  A  G  S  G  E  F
     6
     >>>>>>>>>>>>>>>>>>>>>
     linker

            1810      1820      1830      1840      1850      1860      1870      1880      1890      1900
         *    *    *    *    *    *    *    *    *    *    *    *    *    *    *    *    *    *    *    *
1801 CAGAAGCAGCAGGAAAGACAAAAGAGGGTGTTCTCTATGTAGGCTCCAAAACCAAGGAGGGAGTGGTGCATGGTGTGacaACAGTGGCTGAGAAGACCAA 1900
       E  A  A  G  K  T  K  E  G  V  L  Y  V  G  S  K  T  K  E  G  V  V  H  G  V  T  T  V  A  E  K  T  K
     28
     >>>>>>>>>>>>>>>>>>>>>>>>>>>>>>>>>>>>>>>>>>>>>>>>>>>>>>>>>>>>>>>>>>>>>>>>>>>>>>>>>>>>>>>>>>>>>>>>>>>>
     A53T-?-syn

            1910      1920      1930      1940      1950      1960      1970      1980      1990      2000
```

```
         *    *    *    *    *    *    *    *    *    *    *    *    *    *    *    *    *    *    *    *
1901 AGAGCAAGTGACAAATGTTGGAGGAGCAGTGGTGACGGGTGTGACAGCAGTAGCCCAGAAGACAGTGGAGGGAGCAGGGAGCATTGCAGCAGCCACTGGC 2000
      E  Q  V  T  N  V  G  G  A  V  V  T  G  V  T  A  V  A  Q  K  T  V  E  G  A  G  S  I  A  A  A  T  G
     61
     >>>>>>>>>>>>>>>>>>>>>>>>>>>>>>>>>>>>>>>>>>>>>>>>>>>>>>>>>>>>>>>>>>>>>>>>>>>>>>>>>>>>>>>>>>>>>>>>>>>>
     A53T-?-syn

              2010      2020      2030      2040      2050      2060      2070      2080      2090      2100
         *    *    *    *    *    *    *    *    *    *    *    *    *    *    *    *    *    *    *    *
2001 TTTGTCAAAAAGGACCAGTTGGGCAAGAATGAAGAAGGAGCCCCACAGGAAGGAATTCTGGAAGATATGCCTGTGGATCCTGACAATGAGGCTTATGAAA 2100
     F  V  K  K  D  Q  L  G  K  N  E  E  G  A  P  Q  E  G  I  L  E  D  M  P  V  D  P  D  N  E  A  Y  E  M
     94
     >>>>>>>>>>>>>>>>>>>>>>>>>>>>>>>>>>>>>>>>>>>>>>>>>>>>>>>>>>>>>>>>>>>>>>>>>>>>>>>>>>>>>>>>>>>>>>>>>>>>
     A53T-?-syn

              2110      2120      2130      2140      2150      2160      2170      2180      2190      2200
         *    *    *    *    *    *    *    *    *    *    *    *    *    *    *    *    *    *    *    *
2101 TGCCTTCTGAGGAAGGGTATCAAGACTACGAACCTGAAGCCTAAACGCGTGATCCAATCAACCTCTGGATTACAAAATTTGTGAAAGATTGACTGGTATT 2200
                                                        >>>>>>>>>>>>>>>>>>>>>>>>>>>>>>>>>>>>>>>>>>>>>
                                                        WPRE

       P  S  E  E  G  Y  Q  D  Y  E  P  E  A  *
     128
     >>>>>>>>>>>>>>>>>>>>>>>>>>>>>>>>>>>>>>>>>>>>
     A53T-?-syn

              2210      2220      2230      2240      2250      2260      2270      2280      2290      2300
         *    *    *    *    *    *    *    *    *    *    *    *    *    *    *    *    *    *    *    *
2201 CTTAACTATGTTGCTCCTTTTACGCTATGTGGATACGCTGCTTTAATGCCTTTGTATCATGCTATTGCTTCCCGTATGGCTTTCATTTTCTCCTCCTTGT 2300
     >>>>>>>>>>>>>>>>>>>>>>>>>>>>>>>>>>>>>>>>>>>>>>>>>>>>>>>>>>>>>>>>>>>>>>>>>>>>>>>>>>>>>>>>>>>>>>>>>>>>
     WPRE

              2310      2320      2330      2340      2350      2360      2370      2380      2390      2400
         *    *    *    *    *    *    *    *    *    *    *    *    *    *    *    *    *    *    *    *
2301 ATAAATCCTGGTTGCTGTCTCTTTATGAGGAGTTGTGGCCCGTTGTCAGGCAACGTGGCGTGGTGTGCACTGTGTTTGCTGACGCAACCCCCACTGGTTG 2400
     >>>>>>>>>>>>>>>>>>>>>>>>>>>>>>>>>>>>>>>>>>>>>>>>>>>>>>>>>>>>>>>>>>>>>>>>>>>>>>>>>>>>>>>>>>>>>>>>>>>>
     WPRE

              2410      2420      2430      2440      2450      2460      2470      2480      2490      2500
         *    *    *    *    *    *    *    *    *    *    *    *    *    *    *    *    *    *    *    *
2401 GGGCATTGCCACCACCTGTCAGCTCCTTTCCGGGACTTTCGCTTTCCCCCTCCCTATTGCCACGGCGGAACTCATCGCCGCCTGCCTTGCCCGCTGCTGG 2500
     >>>>>>>>>>>>>>>>>>>>>>>>>>>>>>>>>>>>>>>>>>>>>>>>>>>>>>>>>>>>>>>>>>>>>>>>>>>>>>>>>>>>>>>>>>>>>>>>>>>>
     WPRE

              2510      2520      2530      2540      2550      2560      2570      2580      2590      2600
         *    *    *    *    *    *    *    *    *    *    *    *    *    *    *    *    *    *    *    *
2501 ACAGGGGCTCGGCTGTTGGGCACTGACAATTCCGTGGTGTTGTCGGGGAAATCATCGTCCTTTCCTTGGCTGCTCGCCTGTGTTGCCACCTGGATTCTGC 2600
     >>>>>>>>>>>>>>>>>>>>>>>>>>>>>>>>>>>>>>>>>>>>>>>>>>>>>>>>>>>>>>>>>>>>>>>>>>>>>>>>>>>>>>>>>>>>>>>>>>>>
     WPRE

              2610      2620      2630      2640      2650      2660      2670      2680      2690      2700
         *    *    *    *    *    *    *    *    *    *    *    *    *    *    *    *    *    *    *    *
2601 GCGGGACGTCCTTCTGCTACGTCCCTTCGGCCCTCAATCCAGCGGACCTTCCTTCCCGCGGCCTGCTGCCGGCTCTGCGGCCTCTTCCGCGTCTTCGAGA 2700
     >>>>>>>>>>>>>>>>>>>>>>>>>>>>>>>>>>>>>>>>>>>>>>>>>>>>>>>>>>>>>>>>>>>>>>>>>>>>>>>>>>>>>>>>>>>>>>>>
     WPRE

              2710      2720      2730      2740      2750      2760      2770      2780      2790      2800
         *    *    *    *    *    *    *    *    *    *    *    *    *    *    *    *    *    *    *    *
2701 TCTGCCTCGACTGTGCCTTCTAGTTGCCAGCCATCTGTTGTTTGCCCCTCCCCCGTGCCTTCCTTGACCCTGGAAGGTGCCACTCCCACTGTCCTTTCCT 2800
        >>>>>>>>>>>>>>>>>>>>>>>>>>>>>>>>>>>>>>>>>>>>>>>>>>>>>>>>>>>>>>>>>>>>>>>>>>>>>>>>>>>>>>>>>>>>>
        bGH poly(A)

              2810      2820      2830      2840      2850      2860      2870      2880      2890      2900
         *    *    *    *    *    *    *    *    *    *    *    *    *    *    *    *    *    *    *    *
2801 AATAAAATGAGGAAATTGCATCGCATTGTCTGAGTAGGTGTCATTCTATTCTGGGGGGTGGGGTGGGGCAGGACAGCAAGGGGGAGGATTGGGAAGACAA 2900
     >>>>>>>>>>>>>>>>>>>>>>>>>>>>>>>>>>>>>>>>>>>>>>>>>>>>>>>>>>>>>>>>>>>>>>>>>>>>>>>>>>>>>>>>>>>>>>>>>>>>
     bGH poly(A)

              2910      2920      2930      2940      2950      2960      2970      2980      2990      3000
         *    *    *    *    *    *    *    *    *    *    *    *    *    *    *    *    *    *    *    *
2901 TAGCAGGCATGCTGGGGACTCGAGTTAAGGGCGAATTCCCGATAAGGATCTTCCTAGAGCATGGCTACGTAGATAAGTAGCATGGCGGGTTAATCATTAA 3000
     >>>>>>>>>>>>>>>>>>
     bGH poly(A)
```

```
           3010      3020      3030      3040      3050      3060      3070      3080      3090      3100
         *    *    *    *    *    *    *    *    *    *    *    *    *    *    *    *    *    *    *    *
3001 CTACAAGGAACCCCTAGTGATGGAGTTGGccactccctctctgcgcgctcgctcgctcactgaggccgggcgaccaaaggtcgcccgacgcccgggcttt 3100
                              <<<<<<<<<<<<<<<<<<<<<<<<<<<<<<<<<<<<<<<<<<<<<<<<<<<<<<<<<<<<<<<<<<<<<<<<<<
                              ITR

           3110      3120      3130      3140      3150      3160      3170      3180      3190      3200
         *    *    *    *    *    *    *    *    *    *    *    *    *    *    *    *    *    *    *    *
3101 gcccgggcggcctcagtgagcgagcgagcgcgcagccttaattaacctaattcactggccgtcgttttacaacgtcgtgactgggaaaaccctggcgtta 3200
     <<<<<<<<<<<<<<<<<<<<<<<<<<<<<<<<<<<
     ITR

           3210      3220      3230      3240      3250      3260      3270      3280      3290      3300
         *    *    *    *    *    *    *    *    *    *    *    *    *    *    *    *    *    *    *    *
3201 cccaacttaatcgccttgcagcacatccccctttcgccagctggcgtaatagcgaagaggcccgcaccgatcgcccttcccaacagttgcgcagcctgaa 3300

           3310      3320      3330      3340      3350      3360      3370      3380      3390      3400
         *    *    *    *    *    *    *    *    *    *    *    *    *    *    *    *    *    *    *    *
3301 tggcgaatgggacgcgccctgtagcggcgcattaagcgcggcgggtgtggtggttacgcgcagcgtgaccgctacacttgccagcgccctagcgcccgct 3400
                                 >>>>>>>>>>>>>>>>>>>>>>>>>>>>>>>>>>>>>>>>>>>>>>>>>>>>>>>>>>>>>>>>>>>>>>>
                                 M13 ori

           3410      3420      3430      3440      3450      3460      3470      3480      3490      3500
         *    *    *    *    *    *    *    *    *    *    *    *    *    *    *    *    *    *    *    *
3401 cctttcgctttcttcccttcctttctcgccacgttcgccggctttccccgtcaagctctaaatcgggggctccctttagggttccgatttagtgctttac 3500
     >>>>>>>>>>>>>>>>>>>>>>>>>>>>>>>>>>>>>>>>>>>>>>>>>>>>>>>>>>>>>>>>>>>>>>>>>>>>>>>>>>>>>>>>>>>>>>>>>>>
     M13 ori

           3510      3520      3530      3540      3550      3560      3570      3580      3590      3600
         *    *    *    *    *    *    *    *    *    *    *    *    *    *    *    *    *    *    *    *
3501 ggcacctcgaccccaaaaaacttgattagggtgatggttcacgtagtgggccatcgccctgatagacggtttttcgccctttgacgttggagtccacgtt 3600
     >>>>>>>>>>>>>>>>>>>>>>>>>>>>>>>>>>>>>>>>>>>>>>>>>>>>>>>>>>>>>>>>>>>>>>>>>>>>>>>>>>>>>>>>>>>>>>>>>>>
     M13 ori

           3610      3620      3630      3640      3650      3660      3670      3680      3690      3700
         *    *    *    *    *    *    *    *    *    *    *    *    *    *    *    *    *    *    *    *
3601 ctttaatagtggactcttgttccaaactggaacaacactcaaccctatctcggtctattcttttgatttataagggattttgccgatttcggcctattgg 3700
     >>>>>>>>>>>>>>>>>>>>>>>>>>>>>>>>>>>
     M13 ori

           3710      3720      3730      3740      3750      3760      3770      3780      3790      3800
         *    *    *    *    *    *    *    *    *    *    *    *    *    *    *    *    *    *    *    *
3701 ttaaaaaatgagctgatttaacaaaaatttaacgcgaattttaacaaaatattaacgtttataatttcaggtggcatctttcggggaaatgtgcgcggaa 3800

           3810      3820      3830      3840      3850      3860      3870      3880      3890      3900
         *    *    *    *    *    *    *    *    *    *    *    *    *    *    *    *    *    *    *    *
3801 cccctatttgtttatttttctaaatacattcaaatatgtatccgctcatgagacaataaccctgataaatgcttcaataatattgaaaaaggaagagtat 3900
                                                                                                       M
                                                                                                       1
                                                                                                       >>
                                                                                                       AmpR
                                 >>>>>>>>>>>>>>>>>>>>>>>>>>>>>
                                 AmpR promoter

           3910      3920      3930      3940      3950      3960      3970      3980      3990      4000
         *    *    *    *    *    *    *    *    *    *    *    *    *    *    *    *    *    *    *    *
3901 gagtattcaacatttccgtgtcgcccttattcccttttttgcggcattttgccttcctgtttttgctcacccagaaacgctggtgaaagtaaaagatgct 4000
      S  I  Q  H  F  R  V  A  L  I  P  F  F  A  A  F  C  L  P  V  F  A  H  P  E  T  L  V  K  V  K  D  A
     2
     >>>>>>>>>>>>>>>>>>>>>>>>>>>>>>>>>>>>>>>>>>>>>>>>>>>>>>>>>>>>>>>>>>>>>>>>>>>>>>>>>>>>>>>>>>>>>>>>>>>
     AmpR

           4010      4020      4030      4040      4050      4060      4070      4080      4090      4100
         *    *    *    *    *    *    *    *    *    *    *    *    *    *    *    *    *    *    *    *
4001 gaagatcagttgggtgcacgagtgggttacatcgaactggatctcaatagtggtaagatccttgagagttttcgccccgaagaacgttttccaatgatga 4100
     E  D  Q  L  G  A  R  V  G  Y  I  E  L  D  L  N  S  G  K  I  L  E  S  F  R  P  E  E  R  F  P  M  M  S
     35
     >>>>>>>>>>>>>>>>>>>>>>>>>>>>>>>>>>>>>>>>>>>>>>>>>>>>>>>>>>>>>>>>>>>>>>>>>>>>>>>>>>>>>>>>>>>>>>>>>>>
     AmpR

           4110      4120      4130      4140      4150      4160      4170      4180      4190      4200
```

```
        *    *    *    *    *    *    *    *    *    *    *    *    *    *    *    *    *    *    *    *
4101 gcacttttaaagttctgctatgtggcgcggtattatcccgtattgacgccgggcaagagcaactcggtcgccgcatacactattctcagaatgacttggt 4200
       T  F  K  V  L  L  C  G  A  V  L  S  R  I  D  A  G  Q  E  Q  L  G  R  R  I  H  Y  S  Q  N  D  L  V
     69
     >>>>>>>>>>>>>>>>>>>>>>>>>>>>>>>>>>>>>>>>>>>>>>>>>>>>>>>>>>>>>>>>>>>>>>>>>>>>>>>>>>>>>>>>>>>>>>>>>>
     AmpR

            4210      4220      4230      4240      4250      4260      4270      4280      4290      4300
        *    *    *    *    *    *    *    *    *    *    *    *    *    *    *    *    *    *    *    *
4201 tgagtactcaccagtcacagaaaagcatcttacggatggcatgacagtaagagaattatgcagtgctgccataaccatgagtgataacactgcggccaac 4300
      E  Y  S  P  V  T  E  K  H  L  T  D  G  M  T  V  R  E  L  C  S  A  A  I  T  M  S  D  N  T  A  A  N
     102
     >>>>>>>>>>>>>>>>>>>>>>>>>>>>>>>>>>>>>>>>>>>>>>>>>>>>>>>>>>>>>>>>>>>>>>>>>>>>>>>>>>>>>>>>>>>>>>>>>>
     AmpR

            4310      4320      4330      4340      4350      4360      4370      4380      4390      4400
        *    *    *    *    *    *    *    *    *    *    *    *    *    *    *    *    *    *    *    *
4301 ttacttctgacaacgatcggaggaccgaaggagctaaccgcttttttgcacaacatgggggatcatgtaactcgccttgatcgttgggaaccggagctga 4400
     L  L  L  T  T  I  G  G  P  K  E  L  T  A  F  L  H  N  M  G  D  H  V  T  R  L  D  R  W  E  P  E  L  N
     135
     >>>>>>>>>>>>>>>>>>>>>>>>>>>>>>>>>>>>>>>>>>>>>>>>>>>>>>>>>>>>>>>>>>>>>>>>>>>>>>>>>>>>>>>>>>>>>>>>>>
     AmpR

            4410      4420      4430      4440      4450      4460      4470      4480      4490      4500
        *    *    *    *    *    *    *    *    *    *    *    *    *    *    *    *    *    *    *    *
4401 atgaagccataccaaacgacgagcgtgacaccacgatgcctgtagtaatggtaacaacgttgcgcaaactattaactggcgaactacttactctagcttc 4500
       E  A  I  P  N  D  E  R  D  T  T  M  P  V  V  M  V  T  T  L  R  K  L  L  T  G  E  L  L  T  L  A  S
     169
     >>>>>>>>>>>>>>>>>>>>>>>>>>>>>>>>>>>>>>>>>>>>>>>>>>>>>>>>>>>>>>>>>>>>>>>>>>>>>>>>>>>>>>>>>>>>>>>>>>
     AmpR

            4510      4520      4530      4540      4550      4560      4570      4580      4590      4600
        *    *    *    *    *    *    *    *    *    *    *    *    *    *    *    *    *    *    *    *
4501 ccggcaacaattaatagactggatggaggcggataaagttgcaggaccacttctgcgctcggcccttccggctggctggtttattgctgataaatctgga 4600
      R  Q  Q  L  I  D  W  M  E  A  D  K  V  A  G  P  L  L  R  S  A  L  P  A  G  W  F  I  A  D  K  S  G
     202
     >>>>>>>>>>>>>>>>>>>>>>>>>>>>>>>>>>>>>>>>>>>>>>>>>>>>>>>>>>>>>>>>>>>>>>>>>>>>>>>>>>>>>>>>>>>>>>>>>>
     AmpR

            4610      4620      4630      4640      4650      4660      4670      4680      4690      4700
        *    *    *    *    *    *    *    *    *    *    *    *    *    *    *    *    *    *    *    *
4601 gccggtgagcgtgggtctcgcggtatcattgcagcactggggccagatggtaagccctcccgtatcgtagttatctacacgacggggagtcaggcaacta 4700
     A  G  E  R  G  S  R  G  I  I  A  A  L  G  P  D  G  K  P  S  R  I  V  V  I  Y  T  T  G  S  Q  A  T  M
     235
     >>>>>>>>>>>>>>>>>>>>>>>>>>>>>>>>>>>>>>>>>>>>>>>>>>>>>>>>>>>>>>>>>>>>>>>>>>>>>>>>>>>>>>>>>>>>>>>>>>
     AmpR

            4710      4720      4730      4740      4750      4760      4770      4780      4790      4800
        *    *    *    *    *    *    *    *    *    *    *    *    *    *    *    *    *    *    *    *
4701 tggatgaacgaaatagacagatcgctgagataggtgcctcactgattaagcattggtaactgtcagaccaagtttactcatatatactttagattgattt 4800
       D  E  R  N  R  Q  I  A  E  I  G  A  S  L  I  K  H  W  *
     269
     >>>>>>>>>>>>>>>>>>>>>>>>>>>>>>>>>>>>>>>>>>>>>>>>>>>>>>>>>>>
     AmpR

            4810      4820      4830      4840      4850      4860      4870      4880      4890      4900
        *    *    *    *    *    *    *    *    *    *    *    *    *    *    *    *    *    *    *    *
4801 aaaacttcatttttaatttaaaaggatctaggtgaagatcctttttgataatctcatgaccaaaatcccttaacgtgagttttcgttccactgagcgtca 4900
                                                          >>>>>>>>>>>>>>>>>>>>>>>>>>>>>>>>>>>>>>>>>>>>>>>
                                                          ori

            4910      4920      4930      4940      4950      4960      4970      4980      4990      5000
        *    *    *    *    *    *    *    *    *    *    *    *    *    *    *    *    *    *    *    *
4901 gaccccgtagaaaagatcaaaggatcttcttgagatcctttttttctgcgcgtaatctgctgcttgcaaacaaaaaaaccaccgctaccagcggtggttt 5000
     >>>>>>>>>>>>>>>>>>>>>>>>>>>>>>>>>>>>>>>>>>>>>>>>>>>>>>>>>>>>>>>>>>>>>>>>>>>>>>>>>>>>>>>>>>>>>>>>>>
     ori

            5010      5020      5030      5040      5050      5060      5070      5080      5090      5100
        *    *    *    *    *    *    *    *    *    *    *    *    *    *    *    *    *    *    *    *
5001 gtttgccggatcaagagctaccaactctttttccgaaggtaactggcttcagcagagcgcagataccaaatactgtccttctagtgtagccgtagttagg 5100
     >>>>>>>>>>>>>>>>>>>>>>>>>>>>>>>>>>>>>>>>>>>>>>>>>>>>>>>>>>>>>>>>>>>>>>>>>>>>>>>>>>>>>>>>>>>>>>>>>>
     ori
```

```
           5110      5120      5130      5140      5150      5160      5170      5180      5190      5200
         *    *    *    *    *    *    *    *    *    *    *    *    *    *    *    *    *    *    *    *
5101 ccaccacttcaagaactctgtagcaccgcctacatacctcgctctgctaatcctgttaccagtggctgctgccagtggcgataagtcgtgtcttaccggg 5200
     >>>>>>>>>>>>>>>>>>>>>>>>>>>>>>>>>>>>>>>>>>>>>>>>>>>>>>>>>>>>>>>>>>>>>>>>>>>>>>>>>>>>>>>>>>>>>>>>>>>>
     ori

           5210      5220      5230      5240      5250      5260      5270      5280      5290      5300
         *    *    *    *    *    *    *    *    *    *    *    *    *    *    *    *    *    *    *    *
5201 ttggactcaagacgatagttaccggataaggcgcagcggtcgggctgaacggggggttcgtgcacacagcccagcttggagcgaacgacctacaccgaac 5300
     >>>>>>>>>>>>>>>>>>>>>>>>>>>>>>>>>>>>>>>>>>>>>>>>>>>>>>>>>>>>>>>>>>>>>>>>>>>>>>>>>>>>>>>>>>>>>>>>>>>>
     ori

           5310      5320      5330      5340      5350      5360      5370      5380      5390      5400
         *    *    *    *    *    *    *    *    *    *    *    *    *    *    *    *    *    *    *    *
5301 tgagatacctacagcgtgagctatgagaaagcgccacgcttcccgaagggagaaaggcggacaggtatccggtaagcggcagggtcggaacaggagagcg 5400
     >>>>>>>>>>>>>>>>>>>>>>>>>>>>>>>>>>>>>>>>>>>>>>>>>>>>>>>>>>>>>>>>>>>>>>>>>>>>>>>>>>>>>>>>>>>>>>>>>>>>
     ori

           5410      5420      5430      5440      5450      5460      5470      5480      5490      5500
         *    *    *    *    *    *    *    *    *    *    *    *    *    *    *    *    *    *    *    *
5401 cacgagggagcttccagggggaaacgcctggtatctttatagtcctgtcgggtttcgccacctctgacttgagcgtcgatttttgtgatgctcgtcaggg 5500
     >>>>>>>>>>>>>>>>>>>>>>>>>>>>>>>>>>>>>>>>>>>>>>>>>>>>>>>>>>>>>>>>>>>>>>>>>>>>>>>>>>>>>>>>>>>>>>>>>>>>
     ori

           5510      5520      5530      5540      5550      5560      5570      5580      5590      5600
         *    *    *    *    *    *    *    *    *    *    *    *    *    *    *    *    *    *    *    *
5501 gggcggagcctatggaaaaacgccagcaacgcggcctttttacggttcctggccttttgctgcggttttgctcacatgttctttcctgcgttatcccctg 5600
     >>>>>>>>>>>>>>>>>>>>>>>>>>>>>>>>>>>>
     ori

           5610      5620      5630      5640      5650      5660      5670      5680      5690      5700
         *    *    *    *    *    *    *    *    *    *    *    *    *    *    *    *    *    *    *    *
5601 attctgtggataaccgtattaccgcctttgagtgagctgataccgctcgccgcagccgaacgaccgagcgcagcgagtcagtgagcgaggaagcggaaga 5700

           5710      5720      5730      5740      5750      5760      5770      5780      5790      5800
         *    *    *    *    *    *    *    *    *    *    *    *    *    *    *    *    *    *    *    *
5701 gcgcccaatacgcaaaccgcctctccccgcgcgttggccgattcattaatgcagctggcacgacaggtttcccgactggaaagcgggcagtgagcgcaac 5800

           5810      5820      5830      5840      5850      5860      5870      5880      5890      5900
         *    *    *    *    *    *    *    *    *    *    *    *    *    *    *    *    *    *    *    *
5801 gcaattaatgtgagttagctcactcattaggcaccccaggctttacactttatgcttccggctcgtatgttgtgtggaattgtgagcggataacaatttc 5900

           5910      5920      5930      5940
         *    *    *    *    *    *    *    *    *
5901 acacaggaaacagctatgaccatgattacgccagatttaattaagg 5946
```

Supplementary legend Figure 1A:
Entire gene sequence of the plasmid pAAV_CMVie_SynP_NanoLuc-A53T-synuclein-WRPE.
Highlighted text orange: ITR; blue: CMV enhancer/Synapsin promoter; pink: Nanoluc; light green: linker; dark green: A53T_a-synuclein; red: WRPE; light pink: bGH polyA and grey: non-coding.
